# A Highly Thermostable In_2_O_3_/ITO Thin Film Thermocouple Prepared via Screen Printing for High Temperature Measurements

**DOI:** 10.3390/s18040958

**Published:** 2018-03-23

**Authors:** Yantao Liu, Wei Ren, Peng Shi, Dan Liu, Yijun Zhang, Ming Liu, Zuo-Guang Ye, Weixuan Jing, Bian Tian, Zhuangde Jiang

**Affiliations:** 1 Electronic Materials Research Laboratory, Key Laboratory of the Ministry of Education & International Center for Dielectric Research, Xi’an Jiaotong University, Xi’an 710049, China; liuyt8115@stu.xjtu.edu.cn (Y.L.); liudan235@stu.xjtu.edu.cn (D.L.); zhangyj518@mail.xjtu.edu.cn (Y.Z.); mingliu@mail.xjtu.edu.cn (M.L.); zye@sfu.ca (Z.-G.Y.); 2 International Joint Laboratory for Micro/Nano Manufacturing and Measurement Technologies, Xi’an Jiaotong University, Xi’an 710049, China; wxjing@mail.xjtu.edu.cn (W.J.); t.b12@mail.xjtu.edu.cn (B.T.); zdjiang@mail.xjtu.edu.cn (Z.J.); 3 Department of Electronic Engineering, Xi’an University of Technology, Xi’an 710048, China; 4 Department of Chemistry and 4D LABS, Simon Fraser University, Burnaby, BC V5A 1S6, Canada

**Keywords:** screen printing, thermocouple, Seebeck coefficient, thermoelectric response

## Abstract

An In_2_O_3_/ITO thin film thermocouple was prepared via screen printing. Glass additives were added to improve the sintering process and to increase the density of the In_2_O_3_/ITO films. The surface and cross-sectional images indicate that both the grain size and densification of the ITO and In_2_O_3_ films increased with the increase in annealing time. The thermoelectric voltage of the In_2_O_3_/ITO thermocouple was 53.5 mV at 1270 °C at the hot junction. The average Seebeck coefficient of the thermocouple was calculated as 44.5 μV/°C. The drift rate of the In_2_O_3_/ITO thermocouple was 5.44 °C/h at a measuring time of 10 h at 1270 °C.

## 1. Introduction

Many thermal sensors, such as thermocouples [1,2], infrared sensors [3], and optical fiber sensors [4], have been developed for high temperature measurement, especially in turbine engines. To meet the requirements of such extreme environments, a variety of noble metal materials are used to make thermocouples, such as platinum vs. palladium [5,6] and platinum vs. platinum/rhodium alloy [7]. To achieve in-situ temperature detection of hot end members such as turbine blades and vanes, a thin film thermocouple (TFTC) can be directly integrated with component surfaces [7,8,9]. These can obtain stable outputs at lower temperatures [10]. However, they cannot perform well at high temperatures in oxidation environments due to their weak anti-oxidation characteristics. Oxidation can cause instability and degradation of the noble metal materials of the thermocouple [11]. Thereafter, thin film thermocouples with good oxidation resistance and high temperature stability have become important. 

In order to obtain a high thermoelectric output voltage and high temperature thermal stability in engine applications (a high temperature and oxygen atmosphere), many researchers have focused on composite ceramic oxides for thermocouples to achieve measurements at harsh temperatures. Owing to their high output voltage, quick thermo-response, and good anti-oxidation, metal oxide materials, such as ZnO and Al-doped ZnO (AZO), ITO and In_2_O_3_ have been used to make thin film thermocouples for use in extreme environments [12,13]. Among these metal oxide thermocouples, the In_2_O_3_/ITO (95/5) thin film thermocouple is an important temperature sensor and has potential for use at temperatures up to 1200 °C with high thermal output and quick response [13,14]. As a result, the preparation and performance of an In_2_O_3_/ITO thin film thermocouple working over 1200 °C for a long period of time is worth studying. 

In this paper, an In_2_O_3_/ITO thermocouple was prepared via screen printing. Glass powders were used as additives to improve the microstructure and sintering properties of ITO and In_2_O_3_ films. The surface and cross-sectional morphologies of both ITO and In_2_O_3_ films were studied using SEM. The thermoelectric voltages of the In_2_O_3_/ITO thermocouple were measured. The high thermal response of In_2_O_3_/ITO thin film thermocouples was investigated.

## 2. Materials and Methods 

### 2.1. Fabrication of In_2_O_3_/ITO Thin Film Thermocouple

The In_2_O_3_/ITO thin film thermocouple was deposited on an Al_2_O_3_ substrate via screen printing. The Al_2_O_3_ substrate (100 mm × 25 mm × 0.8 mm) was first cleaned with ethyl alcohol and deionized water with an ultrasonic washing machine and then dried for 2 h at 100 °C. ITO and In_2_O_3_ slurries were prepared for screen printing separately. First, the same amounts of terpilenol and ethyl cellulose (10 wt % of powder) additives were mixed with ITO and In_2_O_3_ powders as starting materials, respectively. After pre-mixing of the slurries, glass additives (mixed by CaO and SiO_2_) were added to the slurries separately under continuous stirring for 3 h to obtain uniform precursors. In order to study the effects of glass additives on the oxide films, the ITO and In_2_O_3_ slurries without glass additives were also prepared for comparison. Shadow masks were employed to form different thermocouple electrodes with various dimensions. The width of the ITO and In_2_O_3_ film electrodes was 4 mm, and the hot junction area was 4 × 4 mm. ITO slurry was screen-printed with a surgical blade onto the surface of the substrate using a screen mask, and dried at 100 °C for 1 h. Then, the In_2_O_3_ thin film was prepared using the same process. The processes were repeated three times to obtain thicker films. The samples prepared were annealed at 600 °C for 1 h and then thermally treated at 1250 °C for 1 h in a furnace in air. Copper wires (20 cm) were attached to the ITO and In_2_O_3_ films via silver paste at the cold junction, and dried at 150 °C for 2 h.

### 2.2. Measurements

**Figure 1:**
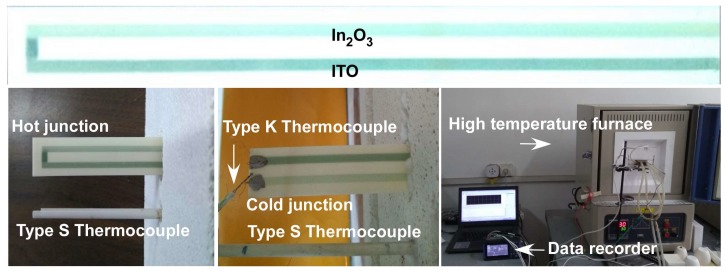
The In_2_O_3_/ITO thermocouple and its measuring system.

SEM surface and cross-sectional images were obtained by a field-emission scanning electron microscope (FESEM, Quanta 250 FEG, FEI, Hillsboro, OR, USA). The thermoelectric response of the In_2_O_3_/ITO thermocouple was obtained by using the lab-made test measurement setup. A schematic diagram of the test system is shown in Figure 1. The hot junction of the thermocouple was placed in a modified high-temperature furnace (KSY-12-16S, Shanghai Laboratory, Shanghai, China), and the cold junction was in the outside of the furnace. The cooling of the cold junction was achieved via natural cooling. The furnace was heated from room temperature (22 °C) to 1270 °C at a heating rate of 5 °C/min. *S*-type and *K*-type thermocouples were used to measure the temperatures of hot sections and cold sections, respectively. The hot junctions of the In_2_O_3_/ITO thermocouple and a standard *S*-type were inserted into the furnace at the same position. The temperature of the cold junction was measured by a standard *K*-type thermocouple. The hot junction temperature (*T_h_*) by *S*-type thermocouple and the cold junction temperature (*T_c_*) by *K*-type thermocouple, as well as the thermoelectric voltage, were recorded at the same time by a data recorder (LR8431, HIOKI Company, Nagano-ken, Japan). The Seebeck coefficient of the thermocouple was calculated and determined accordingly.

## 3. Results and Discussion

### 3.1. Microstructures of ITO and In_2_O_3_ Films

**Figure 2:**
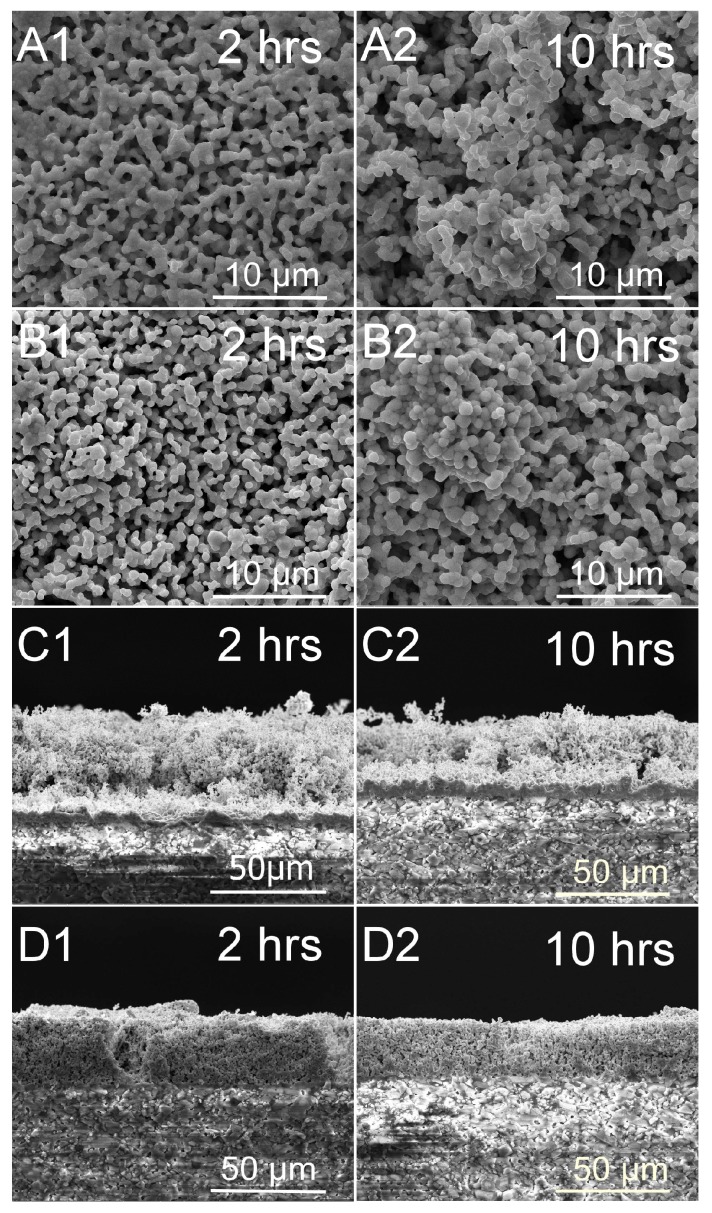
SEM surface and cross-sectional images of ITO and In_2_O_3_ films without glass additives annealed at 1250 °C with different measuring times. (**A**,**C**) ITO film; (**B**,**D**) In_2_O_3_ film.

Figure 2 shows SEM surface and cross-sectional images of ITO and In_2_O_3_ films without glass powder measured at 1250 °C for different times, from 2 to 10 h. The surface images show that both films are very porous and fragile. With the increase in measuring time, the grain size of ITO and In_2_O_3_ films increase. The cross-sectional images indicate that the thickness of the ITO films exhibits a significant decrease from 47.3 µm at 2 h to 34.7 µm at 10 h, while the thickness of the In_2_O_3_ film decreases from 31.5 µm at 2 h to 24.9 µm at 10 h. The results are attributed to the thermal volatilization of the In_2_O_3_/ITO materials under high temperature conditions above 1250 °C [15,16,17], which can lead to deterioration of the high temperature response of the thermocouple and its failure. 

**Figure 3:**
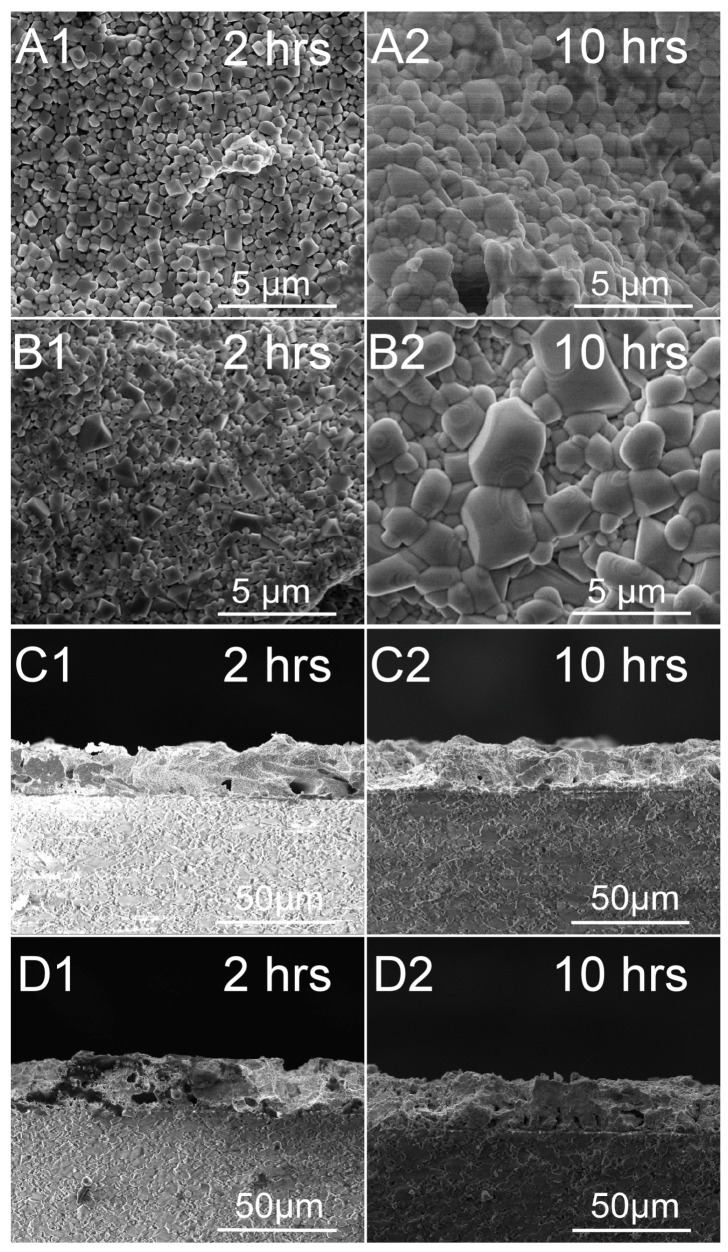
SEM surface and cross-sectional images of ITO and In_2_O_3_ films with 8 wt % glass additives annealed at 1250 °C with different measuring times. (**A**,**C**) ITO film; (**B**,**D**) In_2_O_3_ film.

Figure 3 shows the SEM surface and cross-sectional images of ITO and In_2_O_3_ films with 8 wt % glass additives annealed at 1250 °C with different measuring times, from 2 to 10 h. It can be seen from the surface images that denser films can be obtained for all samples that do not have obvious holes in the film after the addition of the glass additives. The grain size of the ITO and In_2_O_3_ films increases with the annealing time, especially in the In_2_O_3_ films. The cross-sectional images indicate that all films were dense and continuous. There were no significant decreases of the thicknesses for both the ITO and In_2_O_3_ films as measuring time increased. This is attributed to the densification of films after the addition of glass additives, which effectively inhibit the volatilization of ITO and In_2_O_3_. The improved microstructure characteristics therefore lead to a good performance of the In_2_O_3_/ITO thermocouple at high temperature. 

### 3.2. Thermoelectric Properties

**Figure 4:**
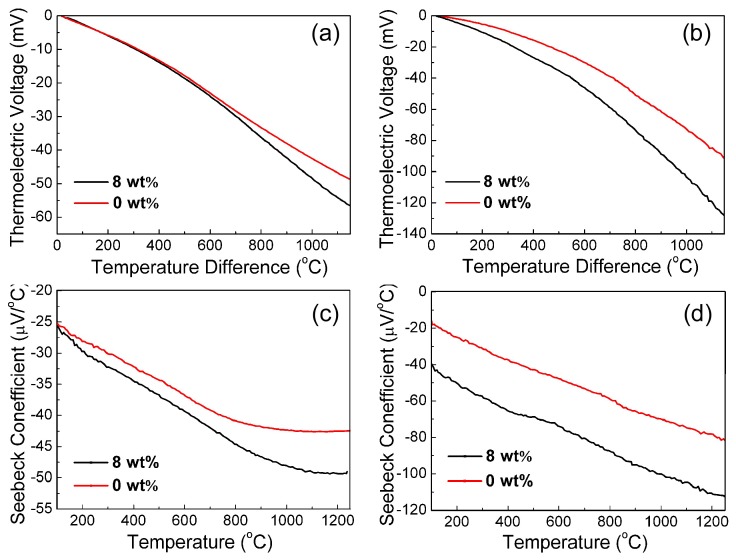
Thermoelectric properties of ITO and In_2_O_3_ films: (**a**,**c**) ITO film; (**b**,**d**) In_2_O_3_ film.

To obtain thermoelectric voltages and the Seebeck coefficient of the ITO and In_2_O_3_ films, pure platinum wires (Seebeck coefficient 1.67 µV/°C [18]) were attached to the ITO or In_2_O_3_ films to form two kinds of ITO–Pt and In_2_O_3_–Pt thermocouples, respectively. As shown in Figure 4, the output voltages and Seebeck coefficients were both negative, due to their *n*-type semiconductor behavior. It can also be seen that, with the increase in temperature to 1150 °C, the absolute value of thermoelectric voltage increased continuously for all samples. For the ITO and In_2_O_3_ films with glass additives, the peak values of voltage could reach up to 56.6 mV and 129.7 mV, respectively, which is much higher than those for the samples without glass powder. From Figure 4c,d, the absolute values of the Seebeck coefficient of the ITO and In_2_O_3_ films with 8 wt % glass additives increased significantly with the increase of temperature difference. The Seebeck coefficients could reach up to 49.2 µV/°C and 112.3 µV/°C, respectively. The cold junction temperature was not stable and increased slowly from room temperature to 81 °C via natural cooling. The cold junction temperature reached 81 °C when the temperature of the hot junction was 1270 °C. The temperature changes in the cold and hot junctions of the thermocouple in the heating process are not shown here, but can be found in the supporting materials Figure S1.

In the heating process of the thermocoples, changes in output voltage can be attributed to the reaction of phonons and electrons. Such interactions are closely dependent on temperature. For non-degenerate semiconductor materials, the Seebeck coefficient is given in Equation (1):

(1) $$S\left(N_{D}\right)=-\frac{Ak}{e}-\frac{k}{e}\ln\left(\frac{{\left(2\pi m_{e}^{*}kT\right)}^{2/3}}{\hbar^{3}N_{D}}\right)$$

where *S* is the Seebeck coefficient, *N_D_* is the carrier concentration, *A* is the transport constant, *k* is the Boltzmann constant, *e* is the electron charge, $m_{e}^{*}$ is the effective mass, and ℏ is Planck’s constant [19]. In a low temperature range, the interactions between phonons and electrons play a major role. The electrons are dragged by phonons along the temperature gradient, resulting in an increase in thermoelectric voltage as temperature difference increases. In a high temperature range, the interactions between phonons and phonons become the main factor, which contributes to the thermal output voltages of the films. At the same time, the electronic flow is inhibited, leading to a slowly increasing Seebeck coefficient, particularly for the ITO films. In addition, the increase in Seebeck coefficient is attributed to the microstructure improved with glass additives, which results in a reduction in charge concentration of the films. 

**Figure 5:**
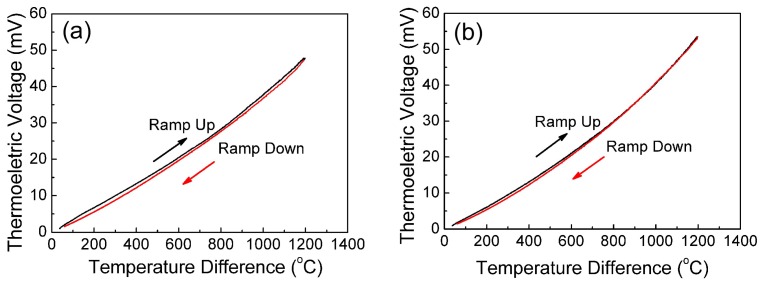
Thermoelectric voltage of In_2_O_3_/ITO thermocouple as a function of temperature difference: (**a**) without glass additives; (**b**) with 8 wt % glass additives.

Figure 5 shows the output thermoelectric voltage of the In_2_O_3_/ITO thermocouple as a function of temperature difference between the hot junction and the cold junction in a heating process. The peak value of the temperature difference can reach 1198 °C. At the same time, the hot juction temperature can reach 1270 °C. To further describe the relationship between thermoelectric voltage and temperature, a third polynomial term is kept in Equation (2) [20]:

(2) $$V\left(\Delta T\right)=A{\left(\Delta T\right)}^{3}+B{\left(\Delta T\right)}^{2}+C\left(\Delta T\right)+D.$$

The coefficients of the polynomial are shown in Table 1, and all *R*^2^ of the values are more than 0.999. The Seebeck coefficient of the thermocouple can be obtained by Equation (3):

(3) $$S=\frac{-\Delta V}{\Delta T}=-\frac{\Delta V_{a}-\Delta V_{b}}{\Delta T}$$

where *S* is the Seebeck coefficient, Δ*T* is temperature difference between the hot junction and the cold junction, Δ*V* is the voltage difference between the ITO and In_2_O_3_ materials, and $\Delta V_{a}$ and $\Delta V_{b}$ is the thermoelectric voltage for the ITO and In_2_O_3_ films, respectively [19,20,21]. The overall Seebeck coefficients are 39.6 µV/°C (without glass additives) and 44.5 µV/°C (with 8 wt % glass additives). 

The Seebeck effect can be attributed to charge–carrier diffusion and phonon drag, which can also be influenced by the microstructure of the electrode materials. The glass additives produce a liquid phase during sintering that can adequately fill the pores between the grains in the ITO and In_2_O_3_ films, obviously increasing the densification of the films with the up to 8% increase in glass additive content. When the content of the additives is over than 8%, the change in densification is not obvious, and the microstructure becomes stable. In addition, the growth of In_2_O_3_ grains is inhibited when too many glass additives are added (SEM pictures are not shown here, but can be found in the supporting materials Figure S2). On the other hand, the charge–carrier concentrations of the films both slightly decrease at the same time, especially for In_2_O_3_ films. The Seebeck coefficient of the In_2_O_3_/ITO thermocouple with a different amount of glass additives at 1270 °C is shown in Table 2. The results show that the Seebeck coefficients of the films slightly increase with the up to 8% increase in glass additives, and the maximum Seebeck coefficient is obtained for the sample with the 8 wt % addition of glass additive.

### 3.3. Thermal Responses of In_2_O_3_/ITO Thin Film Thermocouple

**Figure 6:**
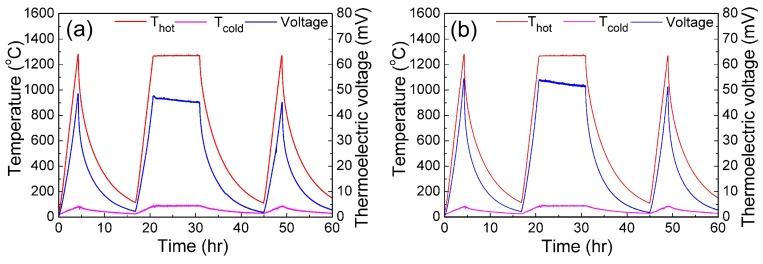
Thermal responses of the thin film thermocouples: (**a**) without glass additives; (**b**) with 8 wt % glass additives.

Figure 6 shows the thermoelectric responses of the In_2_O_3_/ITO thin film thermocouple. There are three thermal cycles in the measurement process. The first and third cycles just show the thermal response for the different heating temperatures. The second cycle shows the thermal stability at high temperature, while the hot juction is holding at a temperature of 1270 °C for 10 h during the measurement process. The performance of thermal response of the thin films thermocouples can be described by the drift rate (*DR*), which is defined according to Equation (4):

(4) $$DR\left(T\right)=\frac{\Delta V\left(T\right)}{V{\left(T\right)}_{ref}}\times \frac{T}{\Delta t}$$

where *DR(T)* is the drift rate, ΔV(T) is the change in voltage at holding temperature, $V{\left(T\right)}_{ref}$ is the initial voltage when the hot junction reaches the maximum temperature, *T* is the temperature of the hot junction, and Δt is the holding time [22]. The drift rate of the In_2_O_3_/ITO thermocouples with 0 and 8 wt % glass additives are 6.81 °C/h and 5.44 °C/h, respectively, as shown in Table 1. By stabilizing the microstructure of the film, the drift rate can be significantly reduced. In addition, the drifting rate seems to reduce significantly after 45 h of measurement, evidenced by the third voltage peak in Figure 6, not significantly lower than the second one for both samples. This phenomena can be attributed to the stabilization of microstructure also. The 8 wt % glass additives make the ITO and In_2_O_3_ films denser, enhance the sintering behavior, lead to the changes in the microstructure of films, and stabilize the characteristics of thermoelectric output. From Figure 6b, the thermoelectric voltage is 51.74 mV at the end of the second cycle. The Seebeck coefficient of the In_2_O_3_/ITO thermocouple is calculated as 43.6 µV/°C at the end of the second cycle. In addition, it can be seen in the third thermal cycle that the thin film thermocouple shows excellent response even after a 10 h measuring time in the second cycle.

## 4. Conclusions

An In_2_O_3_/ITO thin film thermocouple has been prepared on an Al_2_O_3_ substrate via screen printing. After adding 8 wt % glass powder additives, the In_2_O_3_/ITO thin film thermocouple becomes dense and compact, and the evaporation of ITO and In_2_O_3_ film is significantly inhibited. By stabilizing the microstructure of the film, the thermoelectric output voltage and reduction in drift rate for the thermocouple with 8 wt % glass powder additives are both significantly improved. The In_2_O_3_/ITO thin film thermocouple exhibits a high Seebeck coefficient of 44.5 µV/°C and a small drift rate of 5.44 °C/h for a measuring time of 10 h at temperatures as high as 1270 °C. 

## Figures and Tables

**Table 1:** Polynomials used to describe the thermoelectric output of In_2_O_3_/ITO thermocouples with different glass additives.

In_2_O_3_ vs ITO Thermocouples	$V\left(\Delta T\right)=A{\left(\Delta T\right)}^{3}+B{\left(\Delta T\right)}^{2}+C\left(\Delta T\right)+D$				Drift Rate (°C/h)
	*A* (mV/°C^3^)	*B* (mV/°C^2^)	*C* (mV/°C)	*D* (mV)	
0wt %	7.93 × 10^−9^	−4.08 × 10^−6^	0.033	0	6.81
8wt %	7.53 × 10^−9^	2.76 × 10^−6^	0.030	0	5.44

**Table 2:** Seebeck coefficients of In_2_O_3_/ITO thermocouples with different glass additives measured at 1270 °C.

In_2_O_3_/ITO	0 wt %	4 wt %	8 wt %	12 wt %
Seebeck coefficient of In_2_O_3_/ITO at 1270 °C (µV/^o^C)	39.6	41.0	44.5	38.2

## Supplementary Materials

The following are available online at http://www.mdpi.com/1424-8220/18/4/958/s1, Figure S1: The temperature changes of the cold and hot junctions of thermocouple in the heating process, Figure S2: SEM surface images of ITO and In_2_O_3_ films with different glass additives annealed at 1250 °C for 2 h. (A) ITO film, (B) In_2_O_3_ film.
