# Supplementary material for: A Highly Thermostable In2O3/ITO Thin Film Thermocouple Prepared via Screen Printing for High Temperature Measurements
Source: Sensors (Basel). 2018 Mar 23;18(4):958. doi: 10.3390/s18040958 (PMC5948799; doi:10.3390/s18040958)
Supplement: Supplementary file 1 [file sensors-18-00958-s001.pdf]

Supplementary materials for

A Highly Thermostable In_2O_3/ITO Thin Film Thermocouple Prepared via Screen Printing for High Temperature Measurements

Yantao Liu [1,3], Wei Ren [1,*], Peng Shi [1,*], Dan Liu[1], Yijun Zhang [1], Ming Liu [1], Zuo−Guang Ye [1,4], Weixuan Jing [2], Bian Tian [2], and Zhuangde Jiang [2]

1 *Electronic Materials Research Laboratory, Key Laboratory of the Ministry of Education & International Center for Dielectric Research, Xi'an Jiaotong University, Xi'an 710049, China*
2 *International Joint Laboratory for Micro/Nano Manufacturing and Measurement Technologies, Xi'an Jiaotong University, Xi'an 710049, China*
3 *Department of Electronic Engineering, Xi'an University of Technology, Xi'an 710048, China*
4 *Department of Chemistry and 4D LABS, Simon Fraser University, Burnaby, BC V5A 1S6, Canada*

* Corresponding authors' E-mail addresses: wren@mail.xjtu.edu.cn and spxjy@mail.xjtu.edu.cn, Tel.: +86-29-82665670

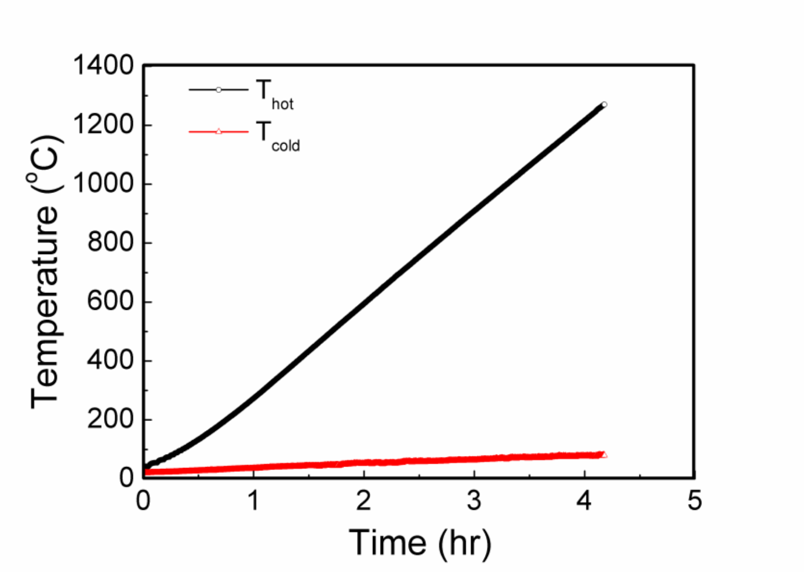

Figure S1. The temperature changes of the cold and hot junctions of thermocouple in the heating process.

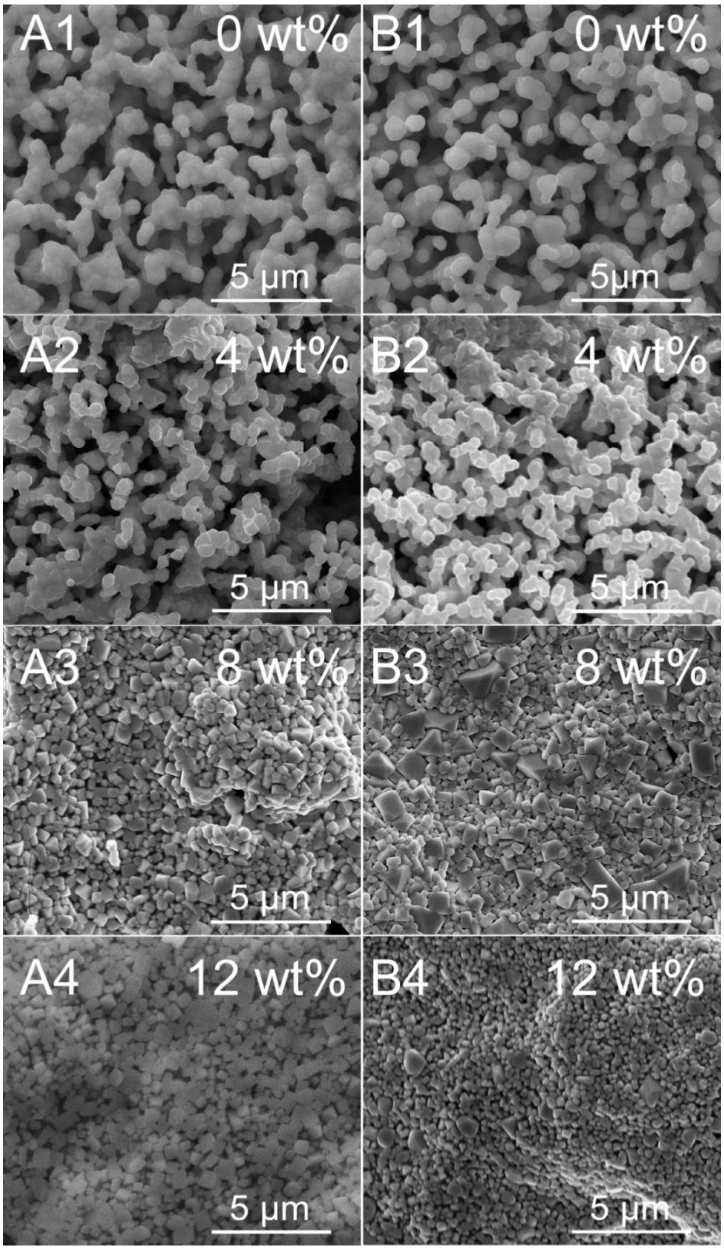

Figure S2. SEM surface images of ITO and In_2O_3 films with different glass additives annealed at 1250 °C for 2 h. (**A**) ITO film, (**B**) In_2O_3 film.
